# Editorial: Balancing Act: Structural-Functional Circuit Disruptions and Compensations in Developing and Aging Brain Disorders

**DOI:** 10.3389/fncir.2019.00083

**Published:** 2020-01-15

**Authors:** Lijun Bai, Tuo Zhang, Xi-Nian Zuo, Mingzhou Ding

**Affiliations:** ^1^The Key Laboratory of Biomedical Information Engineering, Ministry of Education, Department of Biomedical Engineering, School of Life Science and Technology, Xi'an Jiaotong University, Xi'an, China; ^2^School of Automation, Northwestern Polytechnical University, Xi'an, China; ^3^Key Laboratory of Brain and Education, Nanning Normal University, Nanning, China; ^4^Department of Psychology, University of Chinese Academy of Sciences, Beijing, China; ^5^J. Crayton Pruitt Family Department of Biomedical Engineering, University of Florida, Gainesville, FL, United States

**Keywords:** neural circuits, neurobiology, neuroscience, developing and aging brain disorders, structural and functional brain connectivity

The last decade has witnessed an increasing interest in exploring the network connectivity of brain areas and communities. The disruption of brain networks has been linked to variable levels of neuropsychological dysfunctions observed in individual patients with brain disorders (Collin and van den Heuvel, 2013; Crossley et al., 2014). Understanding the course of these changes may help understand how they contribute to risk and resilience for both developing and aging brain disorders, and may offer personalized treatment opportunities. The balancing act of disruptions and compensations in large-scale structural-functional brain network organization across individuals in various brain disorders is still unclear (Bullmore and Sporns, 2009).

The transformative brain changes occurring during the course of childhood and adolescence are critical for the shaping of individual developmental trajectories in cognitive and social functions, adaptability, personality, and mental health (Dosenbach et al., 2010). The tremendous potential for neuroplasticity and environmental sensitivity also characterized this period of development and individualized the brain functional connectome during the course of adolescence and related patterns of maturation (Zielinski et al., 2010; Fair et al., 2012). The progress made on both neuroscience and computational sciences has motivated new approaches for studying brain structure and function from a complex systems perspective (Hagmann et al., 2008; Sharp et al., 2014). These current trends have suggested that connectivity-based methods may provide good tools in order to understand brain functioning in healthy subjects, as well as to study changes during lifespan, or during the time course of neurodegenerative diseases.

Brain connectivity refers to patterns of links connecting distinct units within the nervous system. It can be studied at different scales, and therefore, units or nodes can be defined as individual neurons, neural populations, or segregated brain regions, described by anatomical or functional landmarks. Structural connectivity networks can be measured through white matter tracts quantified by diffusion tractography or correlations of morphological metrics; it can provide clue into structural architectural features. By contrast, functional connectivity networks mainly describe the connective properties of temporal coherences between blood oxygen level-dependent functional MRI signals from both local and distant brain regions; thus functional connectivity networks provide insight of a network perspective on brain dynamics. This Research Topic “Balancing act: structural-functional circuit disruptions and compensations in developing and aging brain disorders” brings together basic, clinical, and translational neuroscience research with brain circuit disruptions and compensations in developing and aging Brain Disorders. The discussions in this Research Topic report new integrated knowledge to understand developing and aging brain disorders.

Neurovascular imbalance is generally noted in the aging population and Alzheimer's disease (AD). It has been shown that regional cerebral blood flow (rCBF) is closely coupled with cerebral metabolism, and the relationship between network measures and rCBF provides insights into the mechanisms of connectivity disruptions in brain disorders. Hu et al. discussed the coupling of rCBF and functional connectivity strength (FCS) in Wilson's disease (WD) associated with mild cognitive impairments (MCI). They found that the CBF-FCS correlations of patients with WD were significantly decreased in the basal ganglia and the cerebellum and slightly increased in the prefrontal cortex and thalamus. Qi et al. evaluated the pattern of activity in the cerebral limbic network from the perspective of the cerebellum. Results indicated that the cerebellum was not compromised by Alzheimer pathology in the early stages of AD, and this pattern indicates that the sub-scale ventral attention network may play a pivotal role in functional compensation through the coupled cerebro-cerebellar limbic network in MCI, and the cerebellum may be a key node in the modulation of social cognition. Moreover, patients with cerebral vascular diseases exhibit widespread differences in functional connectivity across multiple cortical networks. Leukoaraiosis (LA) is associated with cognitive impairment in older people and associated with dysfunctional communications between the three basic brain networks, consisting of the default-mode network (DMN), salience networks (SNs), and the central executive network (CEN). Chen et al. presented the diminished negative correlations between the SN and DMN while positive correlation between the SN and CEN were enhanced as the cognitive impairment loads increased in patients with LA.

Traumatic brain injury (TBI) is a substantial public health problem, and can accelerate the aging process, leading to long-term structural and functional alterations to the brain. Wang et al. aimed to investigate the sex difference on whole-brain functional connectivity at the network level from a cohort of acute mild TBI patients since there were differential cognitive outcome by sex. Ye et al. investigated the changes of α-synuclein in blood serum and its relationship with default mode network (DMN) connectivity after acute mild TBI. The chronic consequences of TBI may contribute to the increased risk for early cognitive decline and dementia, primarily due to diffusion axonal injury. Yin et al. investigated longitudinal changes of white matter (WM) using diffusion tensor imaging (DTI) and their correlations with neuropsychological tests following mild TBI. They reported that increased fractional anisotropy values in some tracts at 1 month post-injury were positively associated with better performance on cognitive information processing speed at initial assessment.

Synaptic failure may critically impair information processing in the brain and may underlie many neurodegenerative diseases. Budak and Zochowski systematically analyzed how two types of synaptic failure (activity-independent and targeted, activity-dependent) affect two complementary (incoming and outgoing) scale-free network structures. Williams and Sun explored the layer and spectrotemporal architecture and laminar distribution of high-frequency oscillations (HFOs) in a neonatal freeze lesion model of focal cortical dysplasia (FCD). They provided the evidence that HFOs, particularly fast ripples, is a biomarker to help define the cortical seizure zone and understand cellular level changes underlying the HFOs. Infarction or aging in regions project to the pyramidal tract (PyT) would result in incomplete transmission of information to the PyT and concomitant decreases in motor planning and coordination abilities. Using the large population data of the HCP and high magnet gradient HARDI data, Wang et al. visualized the existence of the PyT in humans.

Cognitive aging research has identified several general patterns of compensatory neural activity. Most studies of neural compensation limited to a between-subject design, Samuel et al. examined the neural compensation from a fatigue paradigm and reflect neural activities typically associated with aging-related cognitive impairment. In the young cohort, they found that both behavioral performance and neural activity declined as the experiment progressed, reflecting the deleterious effects of cognitive fatigue. Both behavioral performance and neural activity did not decline as the experiment progressed in the older cohort, in contrast to the young. Pelzer et al. reviewed the literature regarding quantitative susceptibility mapping (QSM), MEG, and rs-fMRI detected changes in motor and non-motor symptoms in Parkinson's disease (PD).

Finally, the morphological features of gyri and sulci change during aging and development-related psychiatric disorders such as schizophrenia, reflecting a potential of gyral-sulcal indices as a biomarker for developmental and aging related disorders. Fluid intelligence, as a measure of higher-order relational reasoning, has been argued to be linked to specific functional outcomes and to variations in human neuronal structure and function. Yang et al. explored the temporal variability of cortical gyral-sulcal resting state functional activity and its association with fluid intelligence measures on the Human Connectome Project dataset, which provided novel insights to understand the functional relevance of gyri and sulci. Social anxiety and risk of mental disorders have increased in the left-behind children (LBC). Fu et al. provided empirical evidence of altered brain structure in LBC compared to non-LBC, responsible for emotion regulation and processing, which may account for mental disorders and negative life outcome of LBC.

## Author Contributions

LB and TZ drafted the work and revised it critically for important intellectual content. All of the authors provide approval for publication of the content, agree to be accountable for all aspects of the work in ensuring that questions related to the accuracy or integrity of any part of the work are appropriately investigated and resolved, and made substantial contributions to the conception and design of the work.

### Conflict of Interest

The authors declare that the research was conducted in the absence of any commercial or financial relationships that could be construed as a potential conflict of interest.
